# A ^13^C-NMR Study on the 1,3-Dimethylolurea-Phenol Co-Condensation Reaction: A Model for Amino-Phenolic Co-Condensed Resin Synthesis

**DOI:** 10.3390/polym8110391

**Published:** 2016-11-08

**Authors:** Ming Cao, Taohong Li, Jiankun Liang, Zhigang Wu, Xiaojian Zhou, Guanben Du

**Affiliations:** 1 The Yunnan Province Key Lab of Wood Adhesives and Glued Products, Southwest Forestry University, Kunming 650224, China; caominghappy@swfu.edu.cn (M.C.); liangjiankun@swfu.edu.cn (J.L.); wzghappy@swfu.edu.cn (Z.W.); xiaojianzhou@swfu.edu.cn (X.Z.); 2 College of Materials Science and Engineering, Nanjing Forestry University, Nanjing 210037, China

**Keywords:** co-condensed resin, phenol-urea-formaldehyde, ^13^C-NMR, mechanism

## Abstract

The reactions of di-hydroxymethylurea with phenol under alkaline (pH = 10), weak (pH = 6) and strong acidic (pH = 2) conditions were investigated via the ^13^C-NMR method. Based on the proposed reaction mechanisms, the variations of the structures of different condensed products were analyzed and the competitive relationship between self- and co-condensation reactions was elucidated. The required experimental conditions for co-condensations were clearly pointed out. The main conclusions include: (1) the self-condensation between urea formaldehyde (UF) or phenol formaldehyde (PF) monomers were dominant while the co-condensations were very limited under alkaline conditions. This is because the intermediates produced from urea, methylolurea and phenol are less reactive in co-condensations with respect to self-condensations; (2) under weak acidic conditions, the self-condensations occurred exclusively among the UF monomers. The co-condensation structures were not observed; and (3) the co-condensations became much more competitive under strong acidic conditions as the relative content of the co-condensed methylenic carbon accounts for 53.3%. This result can be rationalized by the high reactivity of the methylolphenol carbocation intermediate toward urea and methylolurea. The revealed reaction selectivity and mechanisms may also be applied to the synthesis of those more complex co-condensed adhesives based on natural phenolic and amino compounds.

## 1. Introduction

Urea-formaldehyde resin (UF) is one of the important wood adhesives, and it is currently the most commonly used amino resin. It is fast curing and affordable, but shows poor water resistance, formaldehyde emission and less stability in storage [1], which limits its applications. Phenol-formaldehyde resin (PF) has excellent bonding strength and water resistance and is applied to the field of outdoor and structural panels. However, there are also disadvantages such as lower curing rate and higher cost [2]. The scholars tried to mix the U, P, and F components in resin synthesis to obtain phenol-urea-formaldehyde co-condensed resin (PUF), aiming to achieve the complementary properties of the two kinds of resins [2,3,4,5,6,7,8,9,10,11,12,13,14]. Nevertheless, copolymer formation depends critically on the competitive relationship between the co-condensation and self-condensation reaction. So far, there are still different opinions in co-condensation reaction conditions. Indeed, even how to identify the co-condensation structures remains a problem.

Currently, the main methods to study the structure of resin are carbon-13 nuclear magnetic resonance (^13^C-NMR) [3,4,5,15,16,17,18,19,20,21,22,23,24,25,26], mass spectrometry (MS) [6,18] and Fourier transform infrared spectroscopy [7,27]. Molecular ion peaks can be obtained in the mass spectrum that is based on soft ionization. These peaks describe the molecular weight of the polymers, but this technology cannot determine whether the co-condensation structures exist because the resin products contain many isomers. In addition to the complex mixture system, the mass spectrometer is basically useless for quantitative analysis of the chemical structures. Information about the chemical groups can be provided in the FT-IR spectra. However, the superposition of signals from different chemical groups may occur in the infrared spectra, and, therefore, this method plays a very limited role in complex mixture analysis. In contrast, the analysis of the structure by ^13^C-NMR is more accurate and allows quantitative analysis of the chemical structure. Therefore, the ^13^C-NMR method is currently the most popular method to study resin structures.

Bunichiro Tomita’s research team described the structure of PUF co-condensation resin [8,9,10]. In their studies, the structure of PUF was analyzed by the ^13^C-NMR method. The chemical shifts at 40–41, 44–45, 46–47 and 49–50 ppm are attributed to the methylene carbons of the co-condensation structures *o*-*Ph*–CH_2_–NHCO–, *p*-*Ph*–CH_2_–NHCO–, *o*-*Ph*–CH_2_–N(–CH_2_–)CO– and *p*-*Ph*–CH_2_–N(–CH_2_–)CO–, respectively(*o-* and *p-* means *ortho-* and *para-*, respecitvely). From then on, similar attributions were proposed by Xianfeng Mo [3], Dongbin Fan [4], Ida Poljansek [7], Guangbo He [11] and Kerstin Schmidt [12]. However, combined with our own earlier study [28] and the results of several reports [3,4,5,8,9,10,11,12], it was found that 44–45 ppm is the only one that can confirm the chemical shifts of the PUF co-condensation structure. This corresponds to the co-condensation structure of *p*-*Ph*–CH_2_–NHCO– because the 40–41 ppm coincides with the chemical shifts in the methylene carbon (*Φ*-CH_2_-*Φ p*–*p*′) in the *para-para* self-condensation bridge bond of phenol-formaldehyde resin, and 46–47 ppm overlaps with the signal of the methylene carbon (–NH–CH_2_–NH–) in unbranched bridges of urea-formaldehyde resin [28]; 49–50 ppm is the same as that of methanol. From the above literature, if the existence of other co-condensation structures was excluded, then the real co-condensation structure content is actually not high in their experiments—it can even be ignored in some experiments. Therefore, the research of the synthetic conditions of the co-condensed resin is directly related to the attribution of the co-condensation structures.

To partially or totally replace the oil-derived chemicals, intensive studies have been carried out to search for non-oil derived substances from renewable resources that can be used in the synthesis of adhesives. A typical representative is tannin, a category of natural phenolic chemicals, which has similar properties to phenol. The studies from Antonio Pizzi’s research team have verified that tannin can be the ideal candidate [23,29,30,31,32,33,34]. To improve the performance of the tannin-based resin, urea was also used as a modifier [35,36,37]. However, analyzing the structure of tannin resin with ^13^C-NMR is much more difficult, due to the complex structure of tannin compounds, as well as the impurity of tannin extraction. Another example is the protein-based adhesives. In protein, the amide structures that are similar to urea exist. To modify the protein adhesives, phenolic compounds were also used, hoping the co-condensation structure can be formed [38]. Similarly, accurate quantitative description of such co-condensed structures is also a tough task. Alternatively, the PUF resin can be viewed as a simpler model. It is very likely that what we found in this study, particularly the revealed reaction selectivity and mechanisms, can also be applied to the synthesis of these co-condensed adhesives based on natural phenolic and amino compounds.

The preparation of PUF resins is generally done by adding phenol during preparation of UF, or adding urea as a modifier in the synthesis of PF, or by using phenol, urea and formaldehyde monomers as raw materials. With the introduction of the third component, it becomes more difficult to study the molecular structures of the resin and that of the reaction processes because the reactions are much more complex. Model compounds can reduce the difficulty in structural analysis. Intuitive information can also be obtained. Therefore, in our study, the reaction system of *N,N*′-di-hydroxymethylurea (UF_2_) and phenol as initial reactants were investigated by employing ^13^C-NMR. The structures of the polymers formed under different conditions were analyzed quantitatively. The competitive relationship between self- and co-condensation reactions and the mechanism of reaction conditions were discussed. The required reaction conditions for the co-condensations were clarified.

## 2. Experimental

### 2.1. Sample Preparation

#### 2.1.1. Samples Prepared under Alkaline and Weak Acidic Conditions

To simulate the formaldehyde aqueous solution (37%), 60 g of UF_2_ (purity > 98%) dissolved in 51 g of water and 9.4 g of phenol were charged into a flask with a stirring device and a condenser. The molar ratio of UF_2_/P was 5:1. The pH was adjusted to 10 and 6 and maintained during the reaction for the alkaline and weak acid condition, respectively. Next, the reaction temperature was gradually increased to 90 °C within 30 min and maintained at 90 °C for 120 min. Sampling was done after the reaction was completed. Samples A1 and A2 were collected at pH 10 and 6, respectively.

#### 2.1.2. Sample Prepared under Strong Acidic Conditions

To simulate the formaldehyde aqueous solution (37%), 12 g of UF_2_ dissolved in 22.91 g of water and 47 g of phenol were charged into a flask with stirring device and condenser. The molar ratio of UF_2_/P was 1/5. The pH value was adjusted to 2. After that, the reaction temperature was gradually raised to 90 °C within 30 min and maintained at 90 °C for 120 min. A sample was collected after the reaction was complete and labeled A3.

### 2.2. ^13^C Nuclear Magnetic Resonance

The ^13^C nuclear magnetic resonance (^13^C-NMR) spectra were measured using a Bruker AVANCE 600 spectrometer (Bruker Corporation, Billerica, MA, USA).

The ^13^C-NMR samples were prepared by dissolving 300 μL of samples in 100 μL of acetone-*d*_6_. The spectra were recorded with a pulse angle of 90 degrees (12 μs) and a relaxation delay of 6 s. To obtain the quantitative results, the inverse-gated decoupling method was applied by using the “zgig” pulse program. The spectra were taken at 150 MHz with 400 accumulated scans. The ^13^C-NMR signals were assigned according to the literature [8,9,10,11,12,15,16,18].

Peaks from methylene carbons were integrated and summed. These were generally less than 100 ppm. The methanol peaks at 50 ppm and methoxyl ether at 56 ppm were exceptions. Finally, the relative contents of all methylene carbons were calculated as the ratio of the integral value of each type of methylene carbon over the total value of all methylene carbons [39]. The carbon atoms in the benzene ring and the carbons of the urea carbonyl group were calculated similarly.

## 3. Results and Discussion

### 3.1. Reactions of UF_2_ and Phenol under Alkaline Conditions

Because thermosetting phenol formaldehyde resin is generally synthesized under alkaline conditions, the reaction medium is advantageous to the self-condensation of PF. With a UF_2_/P molar ratio of 5/1, the low concentrations of phenol cause the self-condensation reaction rate among PF monomers to be small. On the other hand, while the concentration of UF_2_ was high, the self-condensation reaction rate among methylolureas was slow under alkaline conditions. These two factors may allow the co-condensation reactions to participate in the competition. Nevertheless, whether the co-condensation reactions occur still ultimately depends on the competitive relationship among the reactions.

**Figure 1:**
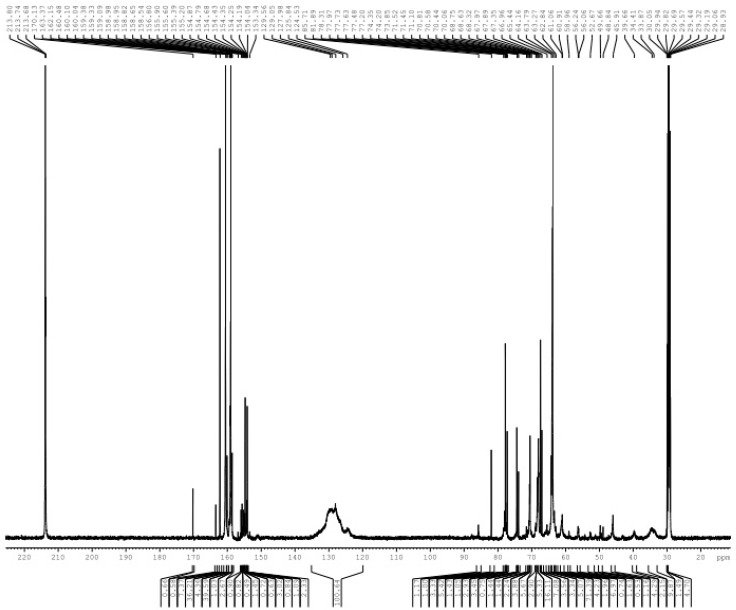
The ^13^C-NMR spectrum of sample A1.

Figure 1 shows the ^13^C-NMR spectrum of sample A1. The chemical structures corresponding to different types of carbons and relative contents for each peak were assigned and listed in Table 1. The signals at 163–164 and 161–162 ppm belong to the carbons in free urea and mono-substituted urea, respectively. This shows that hydrolysis of UF_2_ occurred somewhat in an alkaline environment and released part of the formaldehyde. The total relative content of methylene carbons for the peak at 60–62, 63–65 and 70–72 ppm was up to 57.3%. While the signal of the methylene carbon of the methylol urea overlapped with *p*-methylol phenol at 63–65 ppm, the signal at 70–72 ppm coincided with that of the ether bond methylene carbon formed via condensation of methylol phenol; the concentration of phenolic hydroxyl groups was lower due to the lower relative amount of phenol in the reaction system. Hence, the chemical shifts mainly correspond to the hydroxylmethyl carbon in the methylolurea. The presence of many hydroxylmethyl groups indicated that the reaction rate of UF_2_ condensation is slow under alkaline conditions. Many of the remaining hydroxylmethyl groups did not participate in the condensation reactions.

The methylene bridges and ether bonds of urea were formed in this system. This illustrated that condensation reactions occurred among the hydroxylmethyl products. The relative content of methylene bridge carbons was 4.3%. The relative content of methylene ether bonds without branching (68–70 ppm) was up to 16.7%, which is much higher than the former. Our recent theoretical calculations and experiments confirmed this [28]. The reaction energy barriers of the methylene bridges formed via condensation of UF_2_ were significantly higher than those of the ether bonds. In addition, the formation probability of the bridges was further reduced because the reaction is inhibited by steric hindrance. The signals from the type II and III ether bond methylene carbons (10.7%) may overlap with that of the uron structures at 74–76 and 77–79 ppm [28]. Data from the carbonyl groups show that the relative content of uron structures was 9.0%. Thus, the chemical structures of these two chemical shifts were mostly derived from the uron structures.

The presence of the phenol hydroxylmethyl group indicates that part of the formaldehyde produced by hydrolysis of UF_2_ is transferred to phenol. Therefore, self-condensation reactions of methylolphenol are possible. The relative content of methylene carbons of phenol is 9.8%, which indicates that self-condensation among PF monomers has occurred somewhat. Condensation between *ortho*- and *para*-position (*o*–*p*) was dominant. Compared to the self-condensation products of UF_2_, the self-condensation products of PF are significantly lower due to the low concentration of phenol.

According to the literature [4,21,22,23,24,25], the peaks at 40–41, 44–45, 46–48 and 49–50 ppm were attributed to the methylene carbons of the co-condensation structures *o*-*Ph*–CH_2_–NHCO–, *p*-*Ph*–CH_2_–NHCO–, *o*-*Ph*–CH_2_–N(–CH_2_–)CO– and *p*-*Ph*–CH_2_–N(–CH_2_–)CO–, respectively. Table 1 shows that in addition to the structure represented by the signal at 44–45 ppm, the shift at 40–41 ppm coincided with the chemical shifts of methylene carbons in para-para self-condensation bridges of phenol-formaldehyde resin. The signal at 46–48 ppm overlapped with the methylene carbons of self-condensation structures –NH–CH_2_–NH– (I) of urea-formaldehyde resin. The signal at 49–50 ppm is the same as that of methanol. Therefore, the co-condensation structures could not be determined by the three chemical shifts much less quantitative analysis of their contents. As mentioned, the only one that can confirm the chemical shift of the PUF co-condensation structure is the signal at 44–45 ppm. This corresponded to the co-condensation structure of *p*-*Ph*–CH_2_–NHCO–. However, its relative content is very low in the A1 samples—barely 0.4%.

**Figure 2:**
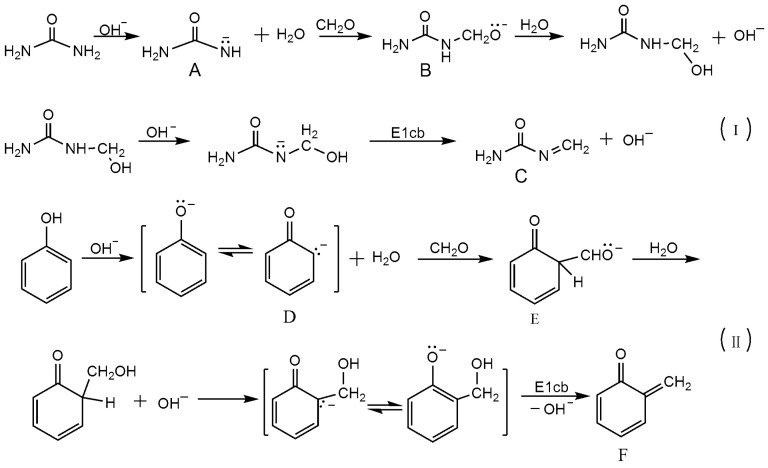
The mechanisms that produce reactive intermediates in the UF (urea formaldehyde) and PF (phenol formaldehyde) reactions under alkaline conditions (pathway I and II, respectively).

**Figure 3:**
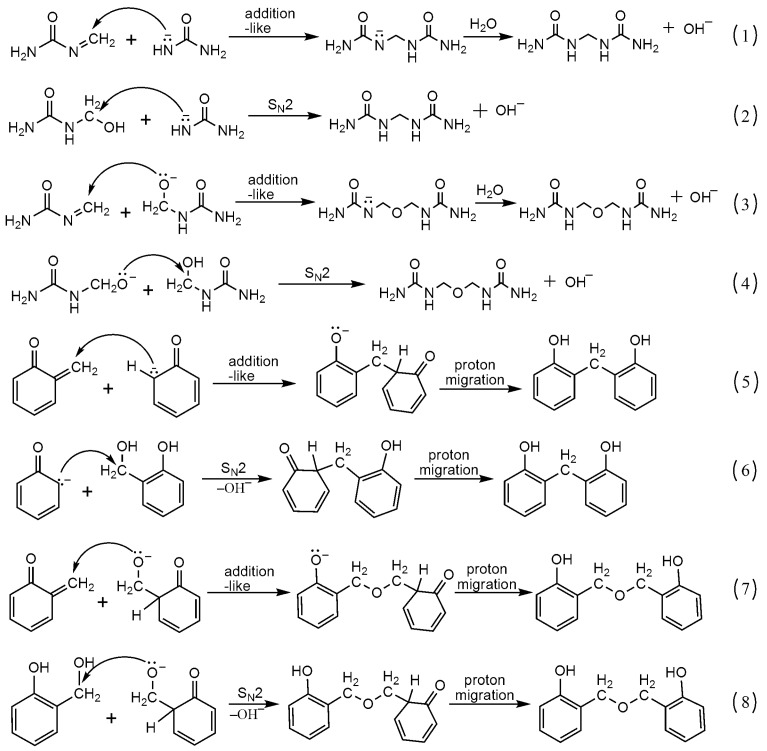
The mechanisms of the UF and PF self-condensation reactions (1)–(8).

The pathway (I) in Figure 2 shows the possible mechanism that forms the reactive intermediates through the reaction between urea and formaldehyde under alkaline conditions. The Species **A**, **B** and **C** are the intermediates that may take part in the following condensation reactions. Among them, the **C** is an oxime-like intermediate that may produced by an E1cb-like loss of OH^−^. Pathway (II) demonstrates the mechanism that forms the intermediates **D**, **E** and **F** for the base-catalyzed phenol-formaldehyde reaction. Among them, the quinine methide is the widely accepted intermediate that is involved in the condensations [40,41,42]. Based on these intermediates, the mechanisms for the UF and PF condensation reactions were proposed in Figure 3. According to the literature, the mechanisms for the UF condensation reactions with alkali catalysis have never been clarified in detail. Using the quinine methide in the PF system for references, the intermediate **C** may be the important one in the UF system. The reaction (1) in Figure 3 shows the proposed addition-like mechanism through which the methylene linkage can be formed. The reaction (2) shows a S_N_2 mechanism that involves the intermediate **A**, a urea anion. The reactions (3) and (4) show the pathways that form ether bonds via the two different mechanisms. It seems that the S_N_2 pathway may be very unlikely because the leaving of OH^−^ may encounter a very high energy barrier. However, our recent theoretical study [28] suggested that such reaction is possible. Because the conjugation effect and intra-molecular hydrogen bonding can stabilize the S_N_2 transition state and lower the energy barrier. The calculated results well rationalized the competitive formation of methylene linkage and ether bonds. Despite this, whether the pathway via oxime-like intermediate is energetically more favorable is unknown. Further detailed theoretical calculations are necessary. Similarly, the quinine methide and S_N_2 mechanisms were shown in Figure 3 as reactions (5)–(8). In fact, the S_N_2 mechanism was also indicated by Conner et al. in their earlier study [43]. However, the two mechanisms are currently still hypotheses. Their competitive relationship remains unclear. Theoretical calculations based on quantum chemistry theory may help us resolve this problem and this work is in the planning stages.

The competitive relationship between co-condensation and self-condensation reactions mainly depends on the relative activity of the intermediates. Based on the ^13^C-NMR data, the highest amount of ether bond structures formed by methylolureas showed that the self-condensations represented by reactions (3) and (4) were in the dominant position. The amount of methylene carbons between phenol is second only to ether bonds, showing that the condensations represented by reactions (5) and (6) significantly contributed to the self-condensation reactions (note that the *ortho*-*para* and *para*-*para* reactions occur via similar mechanism). However, the corresponding self-condensation products were decreased due to the low concentration of phenol. Compared to the self-condensation products, the amount of co-condensed products was very low and illustrated that all of the intermediates exhibited low activity in co-condensations. For a thorough understanding of reaction selectivity, the thermodynamic and kinetic properties of various condensation reactions must be investigated.

### 3.2. Reactions of UF_2_ and Phenol under Weak Acidic Conditions

**Figure 4:**
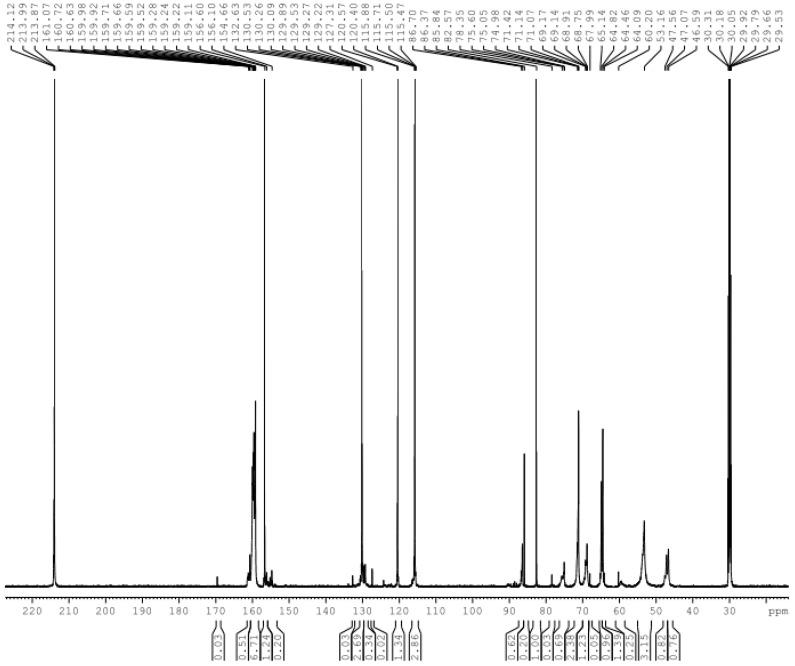
The ^13^C-NMR spectrum of sample A2.

Figure 4 shows the ^13^C-NMR spectrum of sample A2 with analysis in Table 1. There were no self-condensation products of PF monomers formed under the weak acidic conditions. Condensation reactions mainly occurred among methylolurea. In various condensation structures, the number of branched methylene bridges was up to 23.3%. The content was higher than that of the linear bridges (11.6%). Compared to the content under alkaline conditions, the content of linear ether bond structures significantly decreased to 9.4%. The distribution characteristics of these structures are very similar to those obtained from the classical procedure of UF resin synthesis. Although the signal at 46–48 ppm may correspond to the co-condensation structure, there was no corresponding signal at 44 ppm. If the condensation reactions did occur, then the generation probability of linear bridges at 44 ppm is greater than that of branched bridges because the reaction between *N,N*′-dimethylolurea and phenol should mainly produce the linear structure. Consequently, the co-condensation reactions did not occur.

**Figure 5:**
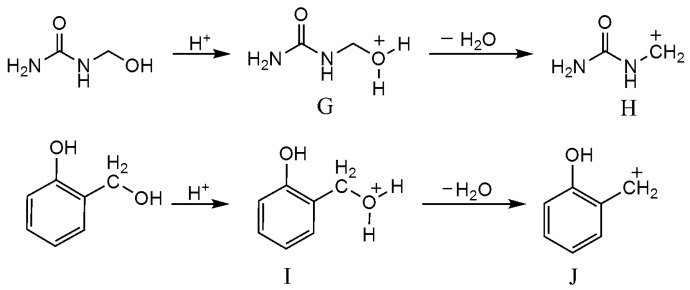
Carboncation formation for UF and PF under acidic condition.

**Figure 6:**
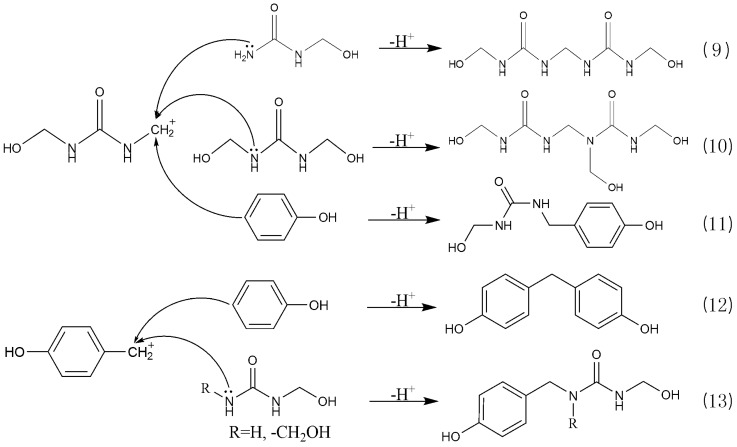
Representative reactions of the PUF_2_ system under acidic conditions (9)–(13).

The intermediates that may be produced under acidic condition were given in Figure 5. For UF reaction, the S_N_2 attack on the protonated intermediate **G** by O or N atom leading to elimination of H_2_O is a possible mechanism. Another mechanism involves the formation of the carboncation intermediate **H** that initiates the following condensation. Such an S_N_1 mechanism has been implied by earlier kinetic experiments [44]. Our theoretical calculations [45] also indicated that the S_N_1 mechanism is very plausible because the carbocation intermediate can be easily formed due to its high stability and the catalytic effect of H_2_O. Similarly, we prefer an S_N_1 mechanism for PF condensation reactions. Based on the carboncations, the proposed self- and co-condensation reactions were given in Figure 6. The carboncation attacked methylolurea to generate a self-condensation bridge structure from the reactions of (9) and (10). Similarly, reaction (11) leads to co-condensation products. However, the formation of co-condensation structure was not observed in the ^13^C-NMR (Figure 4). Therefore, it is believed that the reactivity of the methylolurea carbocation intermediate toward the nitrogen atoms is higher than toward the carbon atoms in the phenol ring. Although there are π electrons on the carbon atoms of phenol and the benzene ring can be activated by the hydroxyl group, nucleophilicity was limited due to a strong electron delocalization effect on the carbons in the large π bond system. Although the lone-paired electrons on the nitrogen atoms in the urea were somewhat delocalized due to the p–π conjugated effect, the results indicate that their nucleophilicity was stronger than that of the carbon atoms on the benzene ring. That is, the nitrogen atoms were selected preferentially when the carbocation intermediates were attacked on nitrogen atoms in urea and carbon atoms in the benzene ring. Thus, only the self-condensation products produced from methylolurea monomers were detected, and there were no co-condensation structures found in sample A2.

Similar to the reactions under alkaline conditions, there were peaks for the methylolphenol (60–62 and 63–65 ppm). Their existence suggested the transfer of formaldehyde produced by hydrolysis of UF_2_. However, the condensation products of phenol were not observed in Figure 4. Therefore, the condensation reactions among PF monomers were difficult under weak acidic conditions. This is because the phenol itself is weakly acidic and the –OH can dissociate protons in solution to form anions. As a result, the effective formation of carboncation intermediate through protonation of methylol group requires higher proton concentration or lower pH. Namely, with pH of 6, the carboncation can not be formed efficiently. The concentration of the anions from dissociation of phenol or methylolphenol in aqueous solution was extremely low, and their activity was much lower than that of the carboncations. Therefore, the contribution of the anion intermediates is ignorable. In other words, the reactions (12) and (13) in Figure 6 barely proceeded. This explains why the self-condensation structures of PF were not seen in sample A2. In general, the self-condensation reactions of PF were avoided under weak acidic conditions. Therefore, it was also difficult to form a competitive relationship between co-condensation reactions and the self-condensation reactions of UF.

### 3.3. Reactions between UF_2_ and Phenol under Strong Acidic Conditions

If the molar ratio *n*(UF_2_):*n*(P) was 5:1 and remained unchanged, then the reaction was particularly aggressive and gelled due to a high concentration of UF_2_. Thus, the molar ratio was adjusted to *n*(UF_2_):*n*(P) = 1:5 with phenol as the main starting material. The presence of large amounts of phenol diluted the UF_2_. The concentration of UF_2_ decreased and reduced the rate of self-condensation. Moreover, the condensation reactions of both UF and PF monomers can occur under strong acidic conditions. Moreover, carbocation intermediates can also be produced from methylolphenol, which are likely involved in the co-condensation reactions. Thus, the competitive relationship between the co-condensation and the self-condensation reactions of PF determines the distribution of product structures.

**Figure 7:**
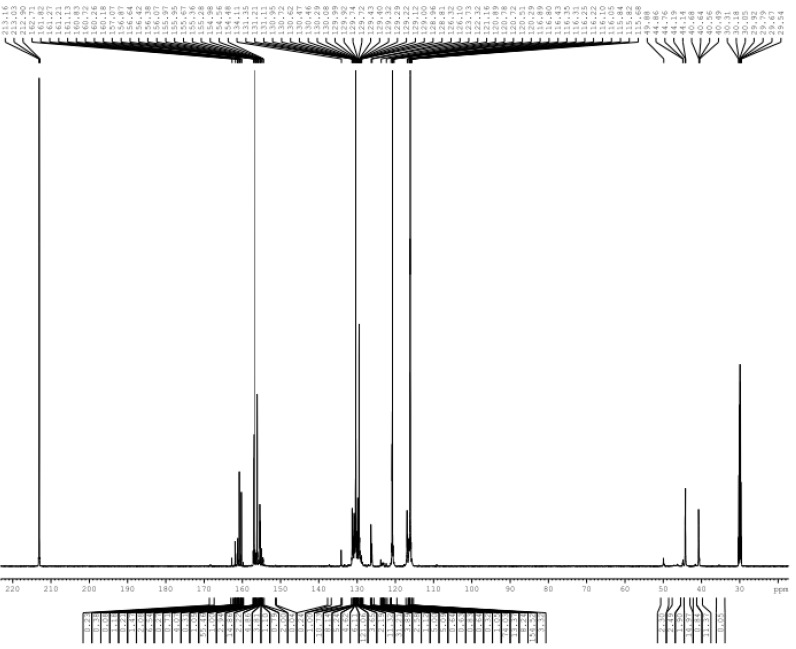
The ^13^C-NMR spectrum of sample A3.

Figure 7 shows the ^13^C-NMR spectrum of sample A3 at pH 2 with quantitative analysis in Table 1. There are no ^13^C-NMR signals of formaldehyde, methylol groups and ether bonds in Figure 7. This confirms that nearly all formaldehyde and methylol groups participated in the formation of condensed structures in the system. This is mainly due to low molar ratio of formaldehyde to urea and phenol (F/(U + P) = 2/(1 + 5)). Alternatively, the nucleophilic centers were far greater than the methylol group. Thus, there were hardly any methylol groups remaining. In addition, the methylene bridges were preferentially generated when the molar ratio was relatively low. A few ether bonds form only when the concentration of methylol group is higher because the thermodynamic stability of the methylene bridges is higher than the ether bonds.

A small amount of self-condensation reactions occurred in UF_2_, and only the linear methylene bridge was generated. The relative content of the corresponding methylene carbon was 7.9%. There were no branched methylene bridges due to the relatively low molar ratio, and a portion of *N,N*′-dimethylolurea was converted to mono-methylolurea or urea in the system.

Only the *p*–*p*′ methylene carbon (40–41 ppm) was generated in the self-condensation productions (38.6%). This is in agreement with the higher relativity of the para position of the phenol compared to the *ortho* position. As shown in Table 1, this chemical shift may also correspond to the co-condensation structure of the *ortho* position of the phenol with methylolurea. Therefore, the relative content might contain the contribution of this structure, but it could not be quantified. According to the assignment of Tomita et al. [8,9,10] the chemical shifts at about 50 ppm may correspond to the co-condensation structure *p*-*Ph*–CH_2_–N(–CH_2_–)CO–. However, the chemical shift of the methyl carbon in methanol is also seen here—the two are overlapped. Although the *N,N*′-dimethylol urea contained hardly any methanol, methanol may be produced from formaldehyde released from methylolureas in alkaline conditions through a Cannizzaro reaction. Thus, there was a signal at 50 ppm in Figure 1, but it could not be determined whether the reaction products in the alkaline condition contain the co-condensation structure. Formaldehyde could not be converted to methanol under acidic conditions, and there was no signal at the expected chemical shift in Figure 4. It seems that the absence of 50 ppm under weak acidic conditions confirms that the signal belongs to methanol. Interestingly, under strong acidic conditions, the signal appeared again. The signal is attributed to the above co-condensation structure after eliminating methanol. However, it was difficult to form the bridges with branched chains because the molar ratio was very low. Thus, the signal is very weak. Although the assignment for co-condensation structures of Tomita et al. [8,9,10] were correct, it was difficult to make a quantitative analysis of the co-condensation structures based on these chemical shifts due to the overlaps. As a result, the evaluation of the reaction conditions for the co-condensation will be unreliable.

As previously described, the chemical shift (44–45 ppm) of the co-condensation structure *p*-*Ph*–CH_2_–NHCO– was not coincident with any other self-condensation structures. Its presence or absence can be a signal that tells if co-condensation occurred or not. In the spectrum of Figure 7, there was a strong signal at the peak corresponding to this methylene carbon whose relative content was up to 53.3%. This was very different from the results under basic and weak acidic conditions. The content of methylene carbon in the co-condensation structures was even more than the sum of that in the self-condensation structures of UF and PF. Such high selectivity under strong acidic conditions suggests that the U/P molar ratio should not be the key factor that affects the competitive relationship between self- and co-condensation reactions.

Under strong acidic conditions, carbocations generated by both methylolurea and methylolphenol (Figure 6) may be involved in the condensation reaction. However, the co-condensation structures were absent in weak acids, indicating that the reaction (11) was difficult. Therefore, we inferred that the co-condensation structures were mainly produced through the reaction of methylolphenol carbocations with urea or methylolurea. This meant that reaction (13) was the true sense of the co-condensation reaction. It is known that the neutral point of phenol solution is around pH = 3, which means that pH that is higher than 3 can not lead to the formation of methylolphenol carboncation intermediates. Therefore, it is very unlikely that the self-condensation reactions of PF monomers or PUF co-condensation reactions would occur efficiently at pH = 4 or 5. Therefore, it should be safe to conclude that the PUF co-condensation reactions may only occur at pH lower than 3.

In summary, under different pH conditions, the competitive relationships between self-condensation and co-condensation reactions were different. This is primarily because of the differences in conditions for the production of reactive intermediates and their reaction activities. When the synthesis conditions are met only for PF or UF in the synthesis of PUF co-condensation resin, the co-condensation reactions cannot proceed or are limited. A high degree of co-condensation can be achieved only when reaction conditions are satisfied simultaneously for UF and PF. Therefore, the true co-condensation resin cannot be obtained when phenol is used to modify the UF resin or the urea is used to modify the PF resin under weak acidic condition. The final resin should be regarded as a blend of both. There were some co-condensation structures produced under weak alkaline conditions, but these were very limited. These conclusions may also be applied to other amino-phenolic co-condensation systems.

However, there were some contradictions and restrictions. On one hand, in strong acid, the condensation reactions were faster than the methylolation reactions for PF. Linear structures were the main condensation products of the polymerization, and the degree of resin branching was low. The resin properties were closer to the thermoplastic resin than the thermosetting resin. Consequently, PF resin modified by a small amount of urea seems difficult to achieve. However, if co-condensation can efficiently occur, the branches in the polymers can be provided by methyolureas. Theoretically, the linear PF polymers can be cross-linked by methylolureas in resin synthesis and form further cross-linking networks in the curing process. On the other hand, if UF was primarily used, it was easy to gel, and the reactions were difficult to control under strong acidic conditions. A practical approach is to use a higher molar ratio of F/U or lower temperature to avoid the gel. That is, modifying the UF resin with a small amount of phenol under strong acidic conditions is feasible. It is likely that the similar situations may also be encountered in the synthesis of the biosourced resins, like tannin and protein resins. In brief, the results and conclusions of this work may be a reference to other amino-phenolic co-condensation systems.

It is worth noting that the co-condensation structures are not only formed in the resin stage—the curing process cannot be ignored. The reactions in the curing process are very different from that in the solution environment. The curing process is performed at a higher temperature, and the water will be eliminated quickly. The system is basically out of the solution environment. Thus, the mobility of the system with a certain molecular weight has been reduced, and functional groups can only approximate the reaction or react in the range of space allowed. In this way, the selectivity due to the difference in reaction activity will be reduced, and there will be more opportunities for the co-condensation reactions to participate in. Of course, these are only inferences based on the results. It is necessary to study the cured structure of resin synthesized under different conditions to make definitive conclusions.

## 4. Conclusions

In this study, the reactions of UF_2_ with phenol under alkaline, weak and strong acidic conditions were investigated by employing ^13^C-NMR. Based on the proposed reaction mechanisms, the variations in the structures of different resins were discussed, and the competitive relationship between self- and co-condensation reactions were elucidated. The required experimental conditions for co-condensations were clearly pointed out.

The self-condensations between UF or PF monomers were dominant while the co-condensations were very limited under alkaline conditions. Under weak acidic conditions, the condensations exclusively occurred among UF monomers, and the co-condensations were not seen. The co-condensations were much more competitive under strong acidic condition because the relative content of the co-condensed methylenic carbon accounted for 53.3%.

The experimental results were explained via the reaction mechanism. Under alkaline conditions, the intermediates produced from urea, methylolurea and phenol are less reactive in co-condensations with respect to self-condensations. The intermediates were mainly formed via methylol urea under weak acidic conditions, and they exhibited low reactivity toward phenol. Thus, the self-condensation reactions among the methylol urea predominate. Under strong acidic condition, the co-condensed structures were mainly resulted from the methylolphenol carboncation which exhibited high reactivity toward the nitrogen atoms in urea or methylolurea. Based on the results of this study, strategies were proposed to improve the procedure of the synthesis of PUF co-condensation resin.

## Figures and Tables

**Table 1:** ^13^C-NMR assignments and the relative content of the methylenic and carbonyl carbons.

Structure	Chemical shift (ppm)	A1 (%)	A2 (%)	A3 (%)	Structure	Chemical shift (ppm)	A1 (%)	A2 (%)	A3 (%)
*Φ*–CH_2_–*Φ o*–*o*′	30–31	3.3	-	-	HO–CH_2_–OH	81–83	0.6	7.4	-
*Φ*–CH_2_–*Φ o*–*p*	33–35	5.3	-	0.2	HOCH_2_–O–CH_2_–OCH_2_OH	85–87	0.6	6.1	-
*Φ*–CH_2_–*Φ p*–*p*′	39–41	1.2	-	38.6	HOCH_2_–O–CH_2_–OCH_2_OH	90–91	-	-	-
*o*-*Ph*–CH_2_–NHCO–					H(CH_2_O)*_n_*OCH_2_OCH_3_	94–95	-	-	-
–NH–CH_2_–NH– (I)	46–48	2.3	11.6	7.9		Total	1.2	13.5	-
*o*-*Ph*–CH_2_–N(–CH_2_–)CO–									
–NH–CH_2_–N= (II)	52–53	1.2	23.3	-	–NH–CH_2_–O–CH_3_	72–73	-	-	-
=N–CH_2_–N= (III)	59–61	0.4	1.7	-	NH_2_–CO–NH_2_	163–164	7.4	-	2.2
*p*-*Ph*–CH_2_–NHCO–	44–45	0.4	-	53.3	NH_2_–CO–NH–	161–162	40.4	-	29.0
*p*-*Ph*–CH_2_–N(–CH_2_–)CO–	49–50	-	-	-	–NH–CO–NH–/–NH–CO–N=	158–160	43.2	97.4	68.8
CH_3_OH ^a^					Uron	154–156	9.0	2.6	-
	Total	14.1	36.6	100.0		Total	100.0	100.0	100.0
–NH–CH_2_OCH_2_NH– (I)	68–70	16.7	9.4	-	Unsubstituted *ortho*	115–119	100.0	39.2	44.1
–NH–CH_2_OCH_2_N= (II)/Uron	74–76	3.0	5.1	-	Unsubstituted *para*	120–124	-	18.4	1.0
=N–CH_2_OCH_2_N= (III)	77–79	7.7	0.2	-	Substituted *ortho*	127–130	-	5.0	16.3
Uron					*meta* Carbon	129–133	-	37.0	38.3
	Total	27.4	14.7		Substituted *para*	132–135	-	0.4	0.3
*o-Ph*-CH_2_OH	60–62	3.8	0.2	-		Total	100.0	100.0	100.0
*p-Ph*-CH_2_OH	63–65	46.4	17.4	-	*ortho* and *para* substitution	151–153	-	-	0.1
–NH–CH_2_OH (I)									
–NH(–CH_2_)–CH_2_OH (II)	70–72	7.1	17.6	-	*ortho* substitution	153–157	72.1	8.0	3.1
*Ph*-CH_2_OCH_2_-*Ph*					*para* substitution	155–158	27.9	92.0	33.0
	Total	57.3	35.2		P	157–158	-	-	63.8
						Total	100.0	100.0	100.0

^a^ CH_3_OH: The methanol signal; the integral was not counted in the quantitative analysis of the methylene carbon.
